# Molecular Profiling of Pediatric *Helicobacter pylori* Gastritis Using Raman Spectroscopy: Spectral Patterns Corresponding to Local Biochemical Changes

**DOI:** 10.1002/jbio.70386

**Published:** 2026-11-06

**Authors:** Jiří Bufka, Lenka Vaňková, Jan Schwarz, Magdalena Daumová, Petr Bouř, Josef Sýkora

**Affiliations:** ^1^ Department of Paediatrics, Faculty of Medicine in Pilsen Charles University in Prague Pilsen Czech Republic; ^2^ Institute of Organic Chemistry and Biochemistry, Czech Academy of Sciences Prague Czech Republic; ^3^ Department of Paediatric and Adult Rheumatology University Hospital Motol Prague Czech Republic; ^4^ Department of Histology and Embryology, Faculty of Medicine in Pilsen Charles University Pilsen Czech Republic; ^5^ Sikl's Department of Pathology, Faculty of Medicine in Pilsen Charles University Pilsen Czech Republic

**Keywords:** biopsy, child, gastric mucosa, gastritis, *Helicobacter pylori*, Raman spectroscopy

## Abstract

*Helicobacter pylori* infection is a major cause of chronic gastritis; however, biomolecular alterations in pediatric gastric mucosa remain poorly characterized. Fresh antral and corpus biopsies from 12 infected children and 20 controls yield 165 Raman spectra at 532 nm. Each baseline‐corrected spectrum undergoes vector normalization before aggregation within site and child. Patient‐level comparisons identify higher relative band areas at 746, 1126, 1300, 1337, 1446, 1583 and 1657 cm^−1^ and lower areas at 1002, 1154, 1515 and 2936 cm^−1^ (all false‐discovery‐rate‐adjusted *p* < 0.05). Statistical significance at 1126, 1583 and 1657 cm^−1^ depends on the normalization range. The bands have overlapping biomolecular assignments and do not establish absolute concentrations, oxidative stress or apoptosis. Raman spectroscopy provides rapid, label‐free molecular fingerprints of pediatric *Helicobacter pylori* gastritis and supports its potential as a translational optical diagnostic approach.

## Graphical abstract

Raman microscopy at 532 nm reveals spectral differences between fresh gastric biopsies from children with *Helicobacter pylori gastritis* and uninfected controls. Patient‐level analysis identifies changes in the relative areas of 11 Raman bands, supporting rapid, label‐free mucosal phenotyping as a potential complement to histopathology.

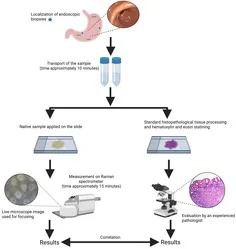

## 1 Introduction

### 1.1 Raman Spectroscopy and Microscopy

Raman spectroscopy (RS) is a non‐destructive analytical technique based on the inelastic scattering of light by molecules. The resulting spectra provide information about molecular vibrations, chemical composition, and structural organization, reflecting the presence of specific chemical groups and biomolecules [1]. In addition to vibrational information, rotational and electronic transitions may also be probed under specific experimental conditions, such as gas‐phase measurements or resonance Raman spectroscopy [2]. A wide range of biological molecules, including carbohydrates, amino acids, nucleic acids, lipids, and carotenoids, can be characterized by RS, with each molecule contributing a characteristic spectral fingerprint [3, 4]. Raman spectra represent the intensity of scattered light as a function of the Raman shift and can be acquired using either Raman spectrometers or Raman microscopes, the latter enabling spatially resolved molecular analysis [1, 2, 3, 4, 5, 6].

Raman spectroscopy and microscopy have been recently explored for many biomedical applications, including imaging of the chemical composition of cells, tissues, and analyzing cellular metabolism [3, 7]. Because of their non‐invasiveness, adaptability and speed of measurement, RS techniques are vigorously explored as potentially useful diagnostic methods, or for investigation of molecular processes in the gastrointestinal system [8, 9, 10, 11].

### 1.2 Gastric Mucosa

The stomach is anatomically divided into the cardia, fundus, body, and pyloric antrum. These regions contain distinct types of gastric mucosa, including cardiac, oxyntic, and antral mucosa [12]. The gastric mucosa consists of a surface epithelium and specialized glands containing multiple cell populations, including mucous, parietal, chief, stem, and neuroendocrine cells [13]. Oxyntic mucosa of the gastric body and fundus is characterized by abundant parietal and chief cells, whereas the antral mucosa contains predominantly mucous cells and a higher density of neuroendocrine G‐ and D‐cells [12, 13, 14].

These regional differences are associated with distinct physiological functions and biochemical compositions, which may contribute to variability in Raman spectral profiles. Therefore, both antral and corpus mucosa were included in the present study.

### 1.3 Pathophysiology of *Helicobacter pylori* Infection

*Helicobacter pylori* is a Gram‐negative bacterium that colonizes the gastric mucosa and is implicated in a broad spectrum of gastroduodenal diseases, including chronic active gastritis, peptic ulcer disease, non‐cardia gastric cancer, and gastric mucosa‐associated lymphoid tissue (MALT) lymphoma. The infection affects approximately half of the global population and is typically acquired during childhood, often persisting throughout life if untreated [15]. Disease outcome results from a complex interplay between bacterial virulence factors, host immune responses, and environmental influences [16, 17]. The pathogenesis of *Helicobacter pylori* infection (Figure 1) can be divided into three major stages: attachment to and colonization of the gastric mucosa, modulation and evasion of host immune responses, and subsequent development of gastric pathology [18]. Following colonization of the gastric mucosa, *Helicobacter pylori* induces chronic inflammation characterized by the release of pro‐inflammatory cytokines, including IL‐8, IL‐1α, IL‐1β, and TNF‐α, together with recruitment of immune cells to the gastric tissue [15, 16, 17, 18]. Key virulence factors, particularly cytotoxin‐associated gene A (cagA) and vacuolating cytotoxin (vacA), contribute to persistent inflammation, immune modulation, and epithelial injury [15, 16, 17].

**FIGURE 1:**
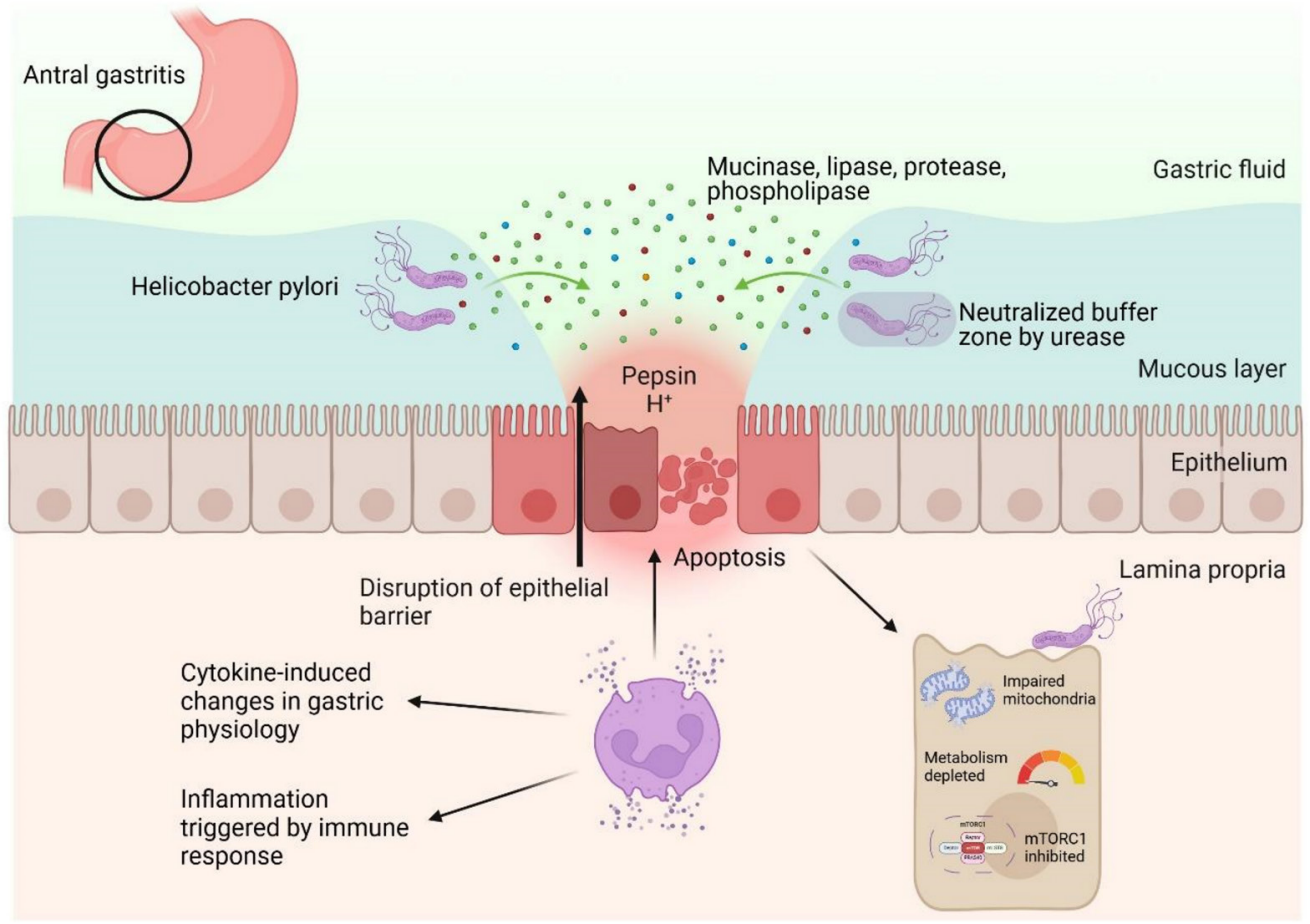
Pathophysiological mechanism of *Helicobacter pylori* infection.

Chronic *Helicobacter pylori* infection is associated with oxidative stress, altered cellular metabolism, increased epithelial turnover, mitochondrial dysfunction, and tissue remodeling, processes that contribute to disease progression and carcinogenesis [19, 20, 21, 22, 23, 24]. These molecular alterations affect proteins, nucleic acids, lipids, and redox‐active molecules, making them potentially detectable by Raman spectroscopy. Understanding these biochemical changes is therefore important for the development of molecular diagnostic approaches and for improving insight into host–pathogen interactions in *Helicobacter pylori* ‐associated disease [16].

#### 1.3.1 Significance of mTORC1 Inhibition in the Pathogenesis of *Helicobacter pylori* Infection

Mechanistic target of rapamycin complex 1 (mTORC1) is a central regulator of cellular metabolism, growth, and immune responses [25]. Experimental studies have shown that *Helicobacter pylori* can modulate mTORC1 signaling, thereby influencing inflammation, cell survival, and host–pathogen interactions [22, 23, 24]. Because mTORC1 is closely linked to mitochondrial function, oxidative stress, and cellular metabolism, its dysregulation may contribute to the pathophysiology of *Helicobacter pylori* ‐associated gastric disease [21, 22, 23, 24, 25]. Targeted modulation of mTORC1 could represent a potential therapeutic approach in the treatment of *Helicobacter pylori* infection and related pathological conditions. mTOR inhibitors, such as rapamycin, have been investigated for their anti‐inflammatory and antitumor effects, but their clinical utility in the context of *Helicobacter pylori* infection requires further investigation [26, 27, 28, 29].

### 1.4 Aims and Hypothesis

To our knowledge, Raman spectroscopic characterization of pediatric gastric mucosa affected by *Helicobacter pylori* infection has not been systematically investigated. Nevertheless, previous studies in adult gastric pathology have demonstrated that Raman spectroscopy can detect molecular alterations associated with inflammation, tissue remodeling, and disease progression. Furthermore, *Helicobacter pylori* infection is known to induce biochemical changes involving proteins, nucleic acids, lipids, oxidative stress pathways, and mitochondrial metabolism, all of which are detectable by Raman spectroscopy. Based on these observations, we hypothesized that infection‐associated molecular alterations in pediatric gastric mucosa would be reflected in characteristic Raman spectral features and could provide candidate molecular signatures associated with disease‐related biochemical alterations.

The primary aim of this study was to establish a methodology for Raman microscopic analysis of freshly obtained gastric biopsies from pediatric patients. The secondary aim was to investigate inter‐individual spectral variability, evaluate changes in relative Raman band intensities and spectral profiles, assign major Raman bands to biologically relevant molecular components, and compare these findings with those obtained from uninfected gastric mucosa.

## 2 Material and Methods

### 2.1 Study Design and Participants

To identify Raman bands possibly useful for diagnosis, Raman spectra of living gastric mucosa cells were collected. The samples were from selected pediatric patients of the Division of Gastroenterology, Department of Paediatrics, University Hospital, Pilsen. The patients (aged 3–18) underwent upper gastrointestinal endoscopy (UGE) from September 2023 to February 2025 and were diagnosed with *Helicobacter pylori* infection. The indications for UGE were persistent gastrointestinal symptoms suggestive of upper digestive tract pathology. Two *Helicobacter pylori* ‐positive patients and two *Helicobacter pylori* ‐negative control subjects were receiving proton pump inhibitor (PPI) therapy at the time of endoscopic examination, reflecting routine clinical practice in children undergoing diagnostic evaluation. No other medications potentially affecting gastric mucosal biochemistry were used. Because PPI therapy may influence gastric physiology, acid secretion, and bacterial density, its potential impact was considered during data interpretation. Visual inspection of Raman spectra did not reveal systematic clustering according to PPI exposure, whereas the dominant spectral differences were associated with *Helicobacter pylori* infection status. However, the present study was not powered to perform a dedicated subgroup analysis of PPI‐treated and untreated patients. Therefore, a possible influence of PPI therapy cannot be completely excluded and is acknowledged as a study limitation.

The endoscopic samples were primarily investigated by classical histological techniques, i.e., no additional stress was put on the patients because of the Raman study. Strict Sydney classification criteria for the patients were applied. Detailed demographic and histopathological data for *Helicobacter pylori* ‐positive patients and healthy controls are presented in Table 1.

**TABLE 1:** Demographic characteristics and histopathological findings according to the modified Sydney classification in *Helicobacter pylori* ‐positive patients and healthy controls.

Parameter	*H. pylori* + (*n* = 12)	Controls (*n* = 20)
Age, years (mean)	8.5	10.6
Male/Female	5/7	12/8
PPI use, *n*	2	2
Modified Sydney classification		
Cellular infiltration (0–5), mean (range)	4.0 (3–5)	0.45 (0–1)
Inflammatory activity (0–5), mean (range)	3.0 (2–4)	0
Atrophy (0–5), mean (range)	1.0 (0–2)	0
Intestinal metaplasia (0–5)	0	0
*H. pylori* density (0–5), mean (range)	3.1 (1–5)	0

### 2.2 Endoscopy, Sample Collection, Handling, Histological Assessment and *Helicobacter pylori* Infection Assay

On entry into the study, all subjects underwent upper gastrointestinal endoscopy (UGE). All UGE examinations were performed by the same experienced pediatric gastroenterologist using a video endoscopy system (GIF XP190N, Olympus, Tokyo, Japan). Informed consent forms were signed by the legal representative prior to endoscopy. During UGE, gastric biopsies were obtained according to the recommendations of the updated Sydney classification. Biopsy specimens were collected from both the gastric antrum and gastric corpus in *Helicobacter pylori* ‐positive patients as well as in *Helicobacter pylori* ‐negative control subjects. Biopsy material from both anatomical regions was used for immediate measurement on a Raman spectrometer (Figure 2). The other gastric samples were fixed in 10% neutral formalin, embedded in paraffin, sectioned at 5 μm, and stained with hematoxylin and eosin (H&E) and Warthin‐Starry for conventional histopathological analysis according to the modified Sydney classification [30]. An additional biopsy from the gastric antrum was used for rapid urease testing. Biopsy specimens were cultured for the presence of *Helicobacter pylori* including the antimicrobial susceptibility. The colonies suspected to be *Helicobacter pylori* were confirmed by microscopic examination and biochemical tests. The biopsy samples were evaluated in a blinded manner by an experienced gastrointestinal pathologist according to the modified Sydney classification. The histopathological findings subsequently underwent an independent review by a second histopathologist to confirm diagnostic consistency.

**FIGURE 2:**
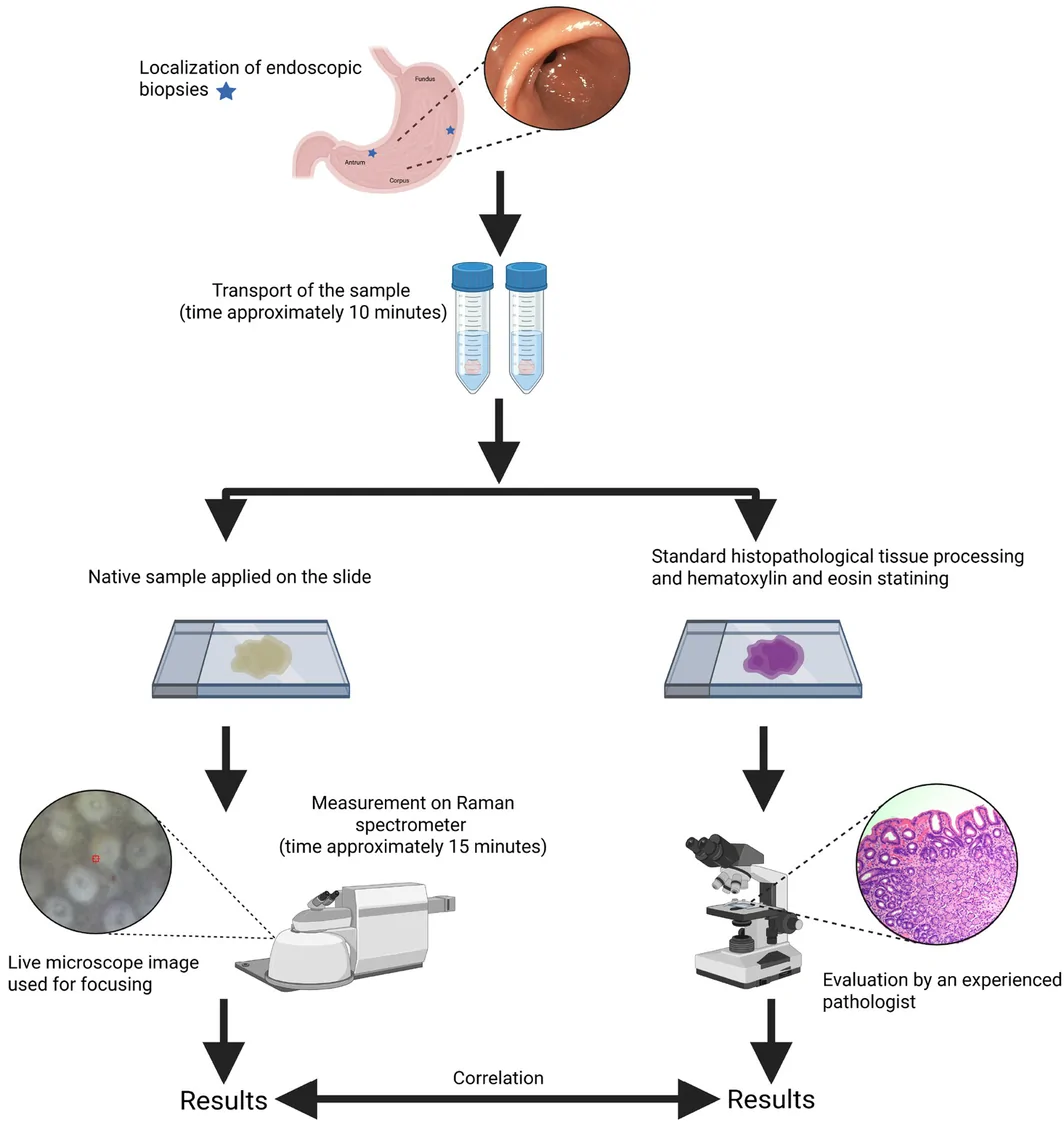
Scheme of the sample preparation for the measurement on Raman spectrometer.

The features of gastric mucosa (the amount and activity of inflammation, lymphocyte and neutrophil granulocyte infiltration, atrophy, metaplasia, hyperplasia, and presence of *Helicobacter pylori* ) were analyzed and graded by the visual analog scale given in the modified Sydney classification, which is the most widely applied clinical protocol [30]. *Helicobacter pylori* infected status was determined by concordance of at least two of three biopsy‐based diagnostic methods: rapid urease testing, histopathological examination, and bacterial culture. Although culture provides excellent specificity and allows antimicrobial susceptibility testing, its sensitivity may be affected by sampling variability, bacterial density, transport conditions, and previous medication exposure. Therefore, a composite diagnostic approach based on concordant results from at least two established methods was used to maximize diagnostic accuracy and minimize false‐negative classification.

### 2.3 Sample Preparation for Raman Microscopy

The biopsy material was put into 0.90% w/v NaCl, anonymized at the time of sampling, immediately transported to the microscopic laboratory, and transferred to a quartz glass slide. The transport time from biopsy to measurement was less than 15 min. If necessary, saline solution was added to prevent the sample from drying out.

### 2.4 Raman Spectroscopy and Data Analysis

Single‐cell Raman spectra were recorded on a Raman microscope (Olympus, Japan) equipped with a spectrometer (DXR1, Thermo Scientific, USA), a 10× objective (Olympus, Japan), a holographic notch filter, charge‐coupled device (CCD) detector, and a laser of wavelength 532 nm and nominal power of 6.5–7 mW (corresponding to ~3 mW at the sample). The signal came from a spot of approximately 50 μm in diameter. Under the laser powers used, no cellular damage was apparent during the measurement. The measurements were finished in less than 30 min from the time of the biopsy. The measured cells were selected using the visual mode of the microscope; the analytical dataset contained two or three spectra per child and anatomical site. Raman spectra were acquired using the single‐point Raman mode. In all experiments, the laser was deliberately focused on the central region of individual gastric epithelial cells. Given this targeting, we assume that the Raman signal originates from both the cell nucleus and the surrounding cytoplasm.

A total of 165 Raman spectra acquired from gastric cells of the antrum and corpus were analyzed, obtained from 12 *Helicobacter pylori* ‐positive patients (five spectra per child; 30 antral and 30 corpus spectra) and 20 healthy control subjects (five or six spectra per child; 53 antral and 52 corpus spectra). Every child contributed to both anatomical sites. Raman spectra were processed using Omnic software (Thermo Scientific, USA). Raman band assignments were further verified using reference spectral libraries from the Institute of Organic Chemistry and Biochemistry, Prague, Czech Republic.

#### 2.4.1 Spectral Preprocessing and Normalization

The raw exports contained 1000 uniformly spaced Raman shifts from 350 to 3100 cm^−1^ (spacing 2.753 cm^−1^). No further interpolation of the spectral axis was applied. Asymmetric least‐squares baseline correction was recomputed from the raw export using a second‐difference penalty, *λ* = 10^6^, asymmetry parameter *p* = 0.001 and 20 iterations, starting with unit weights. In each iteration, weights were set to *p* for intensities above the fitted baseline and to 1 − *p* otherwise. Recomputed residuals agreed with the supplied corrected matrix to within 9.6 × 10^−6^ a.u.; baseline correction was applied only once. No smoothing, additional artifact removal or clipping of negative residuals was performed. All 165 spectra were finite and retained.

Each corrected spectrum y was divided by its Euclidean norm over all 1000 points from 350 to 3100 cm^−1^: y_norm(*νⱼ*) = *y*(*νⱼ*)/‖*y*‖_2_, where ‖*y*‖_2_ is the square root of the sum of squared corrected intensities over that range. Negative residuals were retained in both the norm and subsequent integration. Non‐finite values or a zero normalization factor would stop the analysis; none occurred. Normalization preceded every averaging step, and mean spectra were not normalized again.

The two or three normalized point spectra in each child–site sample were averaged first. Metadata identified one sample per child and site, with no additional biopsy level or repeat visit. For the primary analysis, the antral and corpus means were then averaged with equal weights to obtain one spectrum per child. Both sites were available for all 32 children, so no site imputation was needed. Group means gave each child equal weight regardless of spectrum count. Figures 3 and 4 display site‐specific means with ±1 standard deviation (SD) across children.

**FIGURE 3:**
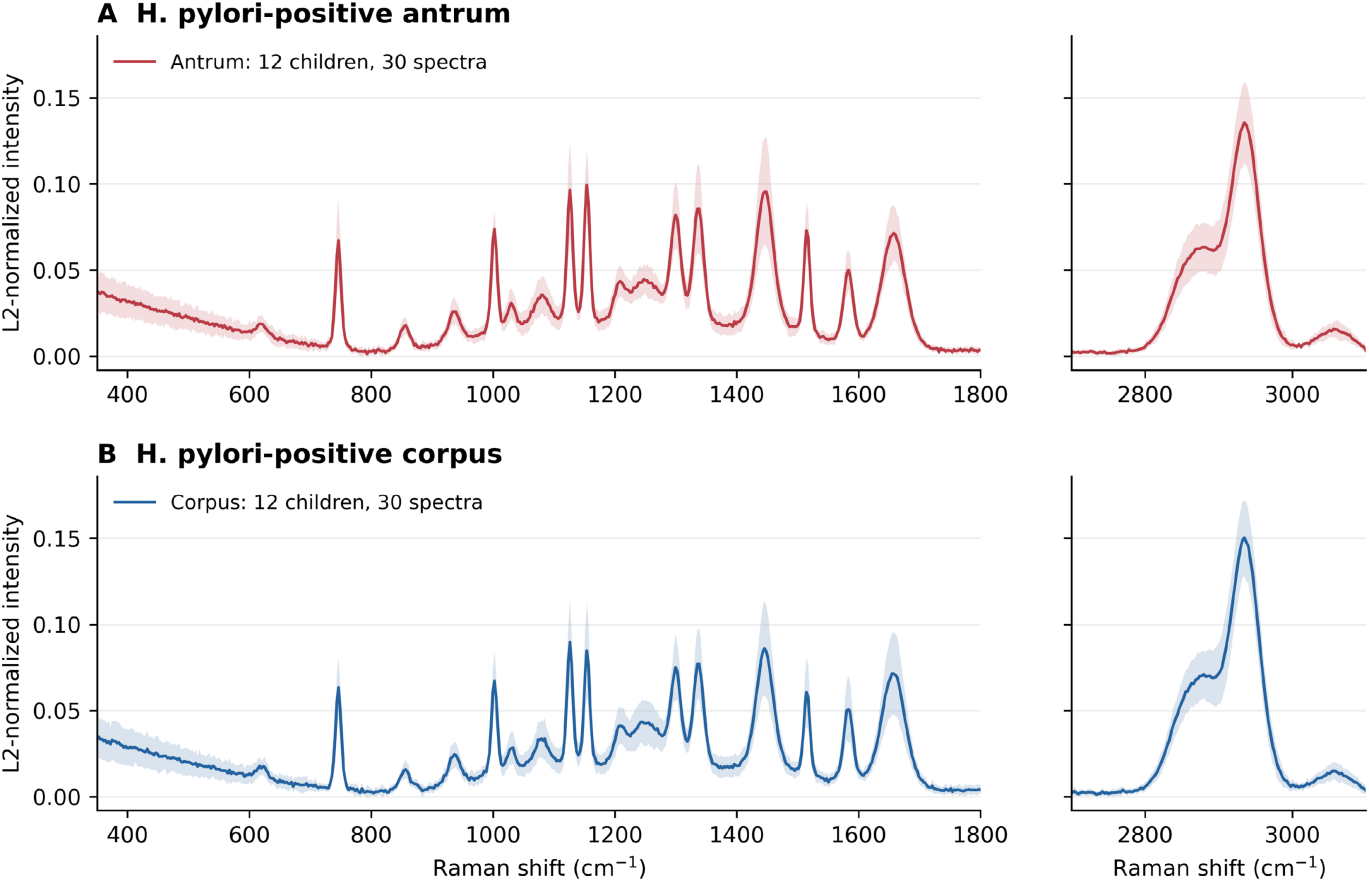
Normalized Raman spectra of *H. pylori* ‐positive gastric samples from (A) antrum and (B) corpus. Solid lines show the mean of 12 child–site mean spectra; shading shows ±1 SD between children. Each site contains 30 point spectra, with two or three spectra per child. Each spectrum underwent ALS baseline correction and L2 normalization over 350–3100 cm^−1^ before averaging within child and site. The displayed ranges are 350–1800 and 2700–3100 cm^−1^; the intervening region is omitted for display only. All panels use the same intensity scale, without vertical offsets or normalization of the mean. Shading represents biological variability, not the standard error or a confidence interval.

**FIGURE 4:**
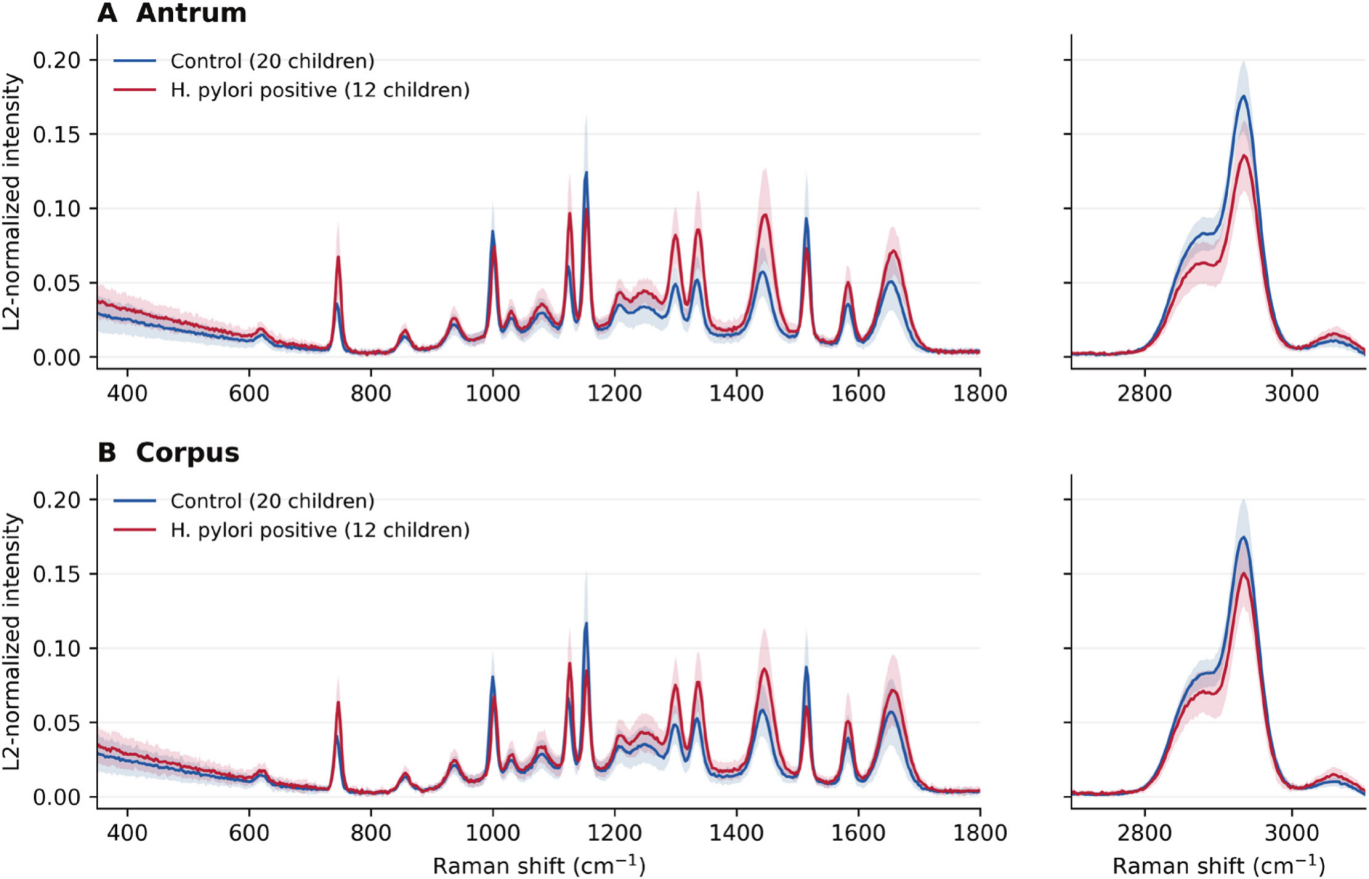
Site‐specific comparison of normalized Raman spectra in controls (blue) and *H. pylori* ‐positive children (red). (A) Antrum: 20 controls/53 spectra and 12 infected children/30 spectra. (B) Corpus: 20 controls/52 spectra and 12 infected children/30 spectra. Solid lines are group means of child–site mean spectra and shaded bands are ±1 SD between children. Every spectrum was ALS‐corrected and L2‐normalized over 350–3100 cm^−1^ before averaging within child and site. Displayed ranges are 350–1800 and 2700–3100 cm^−1^, using a common intensity scale without offsets or additional scaling. Shading does not represent uncertainty of a difference curve; overlap is not a statistical test. Patient‐level inferential results are given in Table 4.

#### 2.4.2 Patient‐Level Statistical Analysis

The independent biological unit was the child (12 infected, 20 controls), not the individual spectrum. Eleven bands named in the submitted manuscript were quantified using fixed integration windows listed in Table 4. Windows were set before the revised group tests, but selection from previously inspected spectra makes this analysis exploratory rather than independently prespecified. Windows were ±10 cm^−1^ around each nominal fingerprint band and ±20 cm^−1^ around 2936 cm^−1^, encompassing several exported grid intervals and the reported small positional variation. Grid spacing was not treated as instrumental spectral resolution. Signed normalized band areas were integrated by the trapezoidal rule with linear interpolation at exact window boundaries.

Between‐group differences in patient mean band areas were evaluated using two‐sided Welch *t*‐tests, targeting differences in means while allowing unequal variances. Mean differences (infected minus control) and unadjusted 95% Welch confidence intervals (CIs) were calculated. The Benjamini–Hochberg (BH) procedure controlled the false discovery rate (FDR) across the family of 11 primary tests; *q* denotes the adjusted *p* value, with *i* < 0.05 considered statistically significant. All 11 results are reported.

Sensitivity analyses compared groups separately at each site (22 tests in one BH family), compared the within‐child antrum‐minus‐corpus contrast between groups (11 tests), averaged all available spectra equally within each child (11 tests), and used vector normalization restricted to exported grid points within 600–1800 cm^−1^ (11 tests). Each latter analysis had its own 11‐test BH family. Python 3.12.14, NumPy 2.3.5, pandas 2.2.3, SciPy 1.17.0 and Matplotlib 3.10.8 were used.

### 2.5 Ethics Statement

Informed consent for the use of the biological material for research purposes was obtained according to the protocol approved by the Ethical Committee, University Hospital Pilsen and the Faculty of Medicine in Pilsen, Charles University, on August 31, 2023, with Reference Number 324/23. The biopsy material from the gastric mucosa was taken endoscopically. The biopsy was provided at the Department of Paediatrics, University Hospital in Pilsen according to the institutional guidelines.

## 3 Results

Raman spectra were measured and compared between 12 patients with *Helicobacter pylori* infection and 20 healthy control subjects, yielding a total of 165 spectra. Both antrum and corpus were available in every child. Figure 3 presents the mean normalized spectra of infected children separately for each site; shading denotes ±1 SD between children. Figure 4 compares infected children and controls at each site. The main patient‐level comparison averaged each child's two site means with equal weights. Tentative biomolecular assignments and the nominal band positions carried forward from the original descriptive analysis are presented in Tables 2 and 3 [31].

**TABLE 2:** Tentative assignments and primary patient‐level changes in normalized band area.

Raman shift (cm^−1^)	Tentative contributors	Primary normalized area
746, 1126	Cytochromes/heme‐associated modes; non‐exclusive	Higher
1300, 1337, 1446	Overlapping nucleic acid, protein and lipid modes; possible cytochrome contributions at 1300 and 1337	Higher
1583, 1657	Protein‐associated regions; possible heme contribution at 1583 and lipid overlap at 1657	Higher
1002, 1154, 1515	Carotenoid‐associated pattern; phenylalanine overlap near 1002	Lower
2936	C–H stretching envelope of lipids and proteins	Lower

**TABLE 3:** Nominal Raman band positions.

Tentative assignment	Control band positions (cm^−1^)	Infected band positions (cm^−1^)
Cytochrome C	744, 1124	746, 1126
Carotenoids	1000, 1153, 1515	1002, 1154, 1515
Nucleic acids	1300, 1335, 1443	1300, 1337, 1446
Proteins	1583, 1654	1583, 1657
C—H stretching (lipids/proteins)	2935 (CH stretch)	2936 (CH stretch)

Compared with controls, infected children had higher normalized band areas at 746, 1126, 1300, 1337, 1446, 1583 and 1657 cm^−1^ and lower areas at 1002, 1154, 1515 and 2936 cm^−1^. All 11 primary comparisons remained significant after BH correction (*q* = 0.000571–0.044091; Table 4). For example, the mean differences were 0.306 (95% CI 0.174–0.439) at 746 cm^−1^, −0.287 (−0.473 to −0.102) at 1515 cm^−1^ and −1.133 (−1.665 to −0.602) at 2936 cm^−1^. These are integrated relative spectral measures, not absolute molecular concentrations. Assignments overlap: heme‐containing molecules, proteins, nucleic acids and lipids can contribute to the same spectral regions, and 532 nm excitation preferentially enhances selected chromophores [32, 33, 34]. No quantitative inference about peak shifts or band broadening was made.

**TABLE 4:** Patient‐level comparison of integrated normalized Raman band areas.

Center/window (cm^−1^)	Infected	Controls	Difference (95% CI)	*p*	*q*
746 736–756	0.776 ± 0.191	0.470 ± 0.136	0.306 (0.174, 0.439)	0.0001	0.0007
1002 992–1012	0.930 ± 0.128	1.054 ± 0.207	−0.124 (−0.245, −0.004)	0.0441	0.0441
1126 1116–1136	1.233 ± 0.258	0.886 ± 0.208	0.348 (0.164, 0.531)	0.0008	0.0018
1154 1144–1164	1.216 ± 0.240	1.557 ± 0.397	−0.340 (−0.570, −0.111)	0.0051	0.0070
1300 1290–1310	1.347 ± 0.282	0.854 ± 0.213	0.493 (0.295, 0.691)	5.19e‐05	0.0006
1337 1327–1347	1.397 ± 0.357	0.877 ± 0.259	0.520 (0.271, 0.768)	0.0003	0.0009
1446 1436–1456	1.689 ± 0.515	1.052 ± 0.294	0.637 (0.291, 0.983)	0.0013	0.0024
1515 1505–1525	0.914 ± 0.190	1.201 ± 0.324	−0.287 (−0.473, −0.102)	0.0036	0.0056
1583 1573–1593	0.827 ± 0.227	0.606 ± 0.222	0.222 (0.052, 0.391)	0.0128	0.0150
1657 1647–1667	1.368 ± 0.361	1.010 ± 0.381	0.358 (0.080, 0.635)	0.0136	0.0150
2936 2916–2956	4.907 ± 0.719	6.040 ± 0.673	−1.133 (−1.665, −0.602)	0.0002	0.0008

*Note:* Values are mean ± SD across children (12 infected; 20 controls). Areas are calculated from dimensionless L2‐normalized intensity integrated over Raman shift (cm^−1^). Each child contributes the equally weighted mean of antrum and corpus. Difference = infected − control; 95% CIs are unadjusted Welch intervals. Tests are two‐sided Welch *t*‐tests; *q* is BH‐adjusted across all 11 primary bands. Nominal centers identify windows, not independently estimated peak positions.

Abbreviations: BH, Benjamini–Hochberg; CI, confidence interval; SD, standard deviation.

Directions of all 11 group differences were consistent in antrum and corpus, although site‐specific BH‐adjusted significance was not reached at 1002 cm^−1^ in either site or at 1583 and 1657 cm^−1^ in corpus (Table S1). No group difference in the paired site contrast remained significant after correction (all *q* ≥ 0.228; Table S2); this does not establish regional equivalence. Averaging all spectra equally within each child preserved all primary directions and significance (Table S3). With 600–1800 cm^−1^ normalization, all directions were retained, but 1126, 1583, and 1657 cm^−1^ were no longer significant (*q* = 0.0880, 0.2286, and 0.2500, respectively; Table S4).

## 4 Discussion

To our knowledge, there are currently no published studies applying Raman microscopy to freshly obtained gastric biopsies from pediatric patients with *Helicobacter pylori* infection. Our Raman spectroscopic analysis identified patient‐level differences in relative normalized band areas in regions tentatively associated with cytochromes, nucleic acids, proteins, carotenoids, and lipids. These spectral associations are compatible with local biochemical alterations in chronic gastritis, but they do not establish the identity or absolute concentration of individual molecules.

The normalized spectral profiles showed variability between children and anatomical sites (Figures 3 and 4). Cellular composition, epithelial injury, inflammatory cell infiltration, tissue remodeling, and bacterial material are possible contributors to this variability. Among the most prominent findings was an increase in normalized band area near 746 and 1126 cm^−1^, compatible with cytochrome‐associated vibrational modes. Cytochrome c is a key component of the mitochondrial electron transport chain and an important mediator of intrinsic apoptotic signaling. Experimental studies have demonstrated that *Helicobacter pylori* can induce oxidative stress and mitochondrial apoptosis in gastric epithelial cells [32, 33, 34, 35]. Raman studies also show that cytochrome signals depend on redox state and resonance enhancement [33, 34, 35, 36, 37]. Our measurements did not determine cytochrome c localization, redox ratios, mitochondrial function, or apoptosis. These mechanisms therefore remain hypotheses, and the 1126 cm^−1^ result was sensitive to the normalization range.

Higher normalized band areas were also observed near 1300, 1337, 1446, 1583 and 1657 cm^−1^. These regions contain overlapping contributions from nucleic acids, proteins, and lipids, and cytochromes may contribute near 1300, 1337, and 1583 cm^−1^ under visible excitation [32, 34]. Consequently, the results cannot demonstrate increased DNA or RNA content or protein synthesis. Chronic inflammation and epithelial regeneration provide plausible biological context, but the molecular source of each difference requires complementary validation. The 1583 and 1657 cm^−1^ differences were not significant with the alternative normalization range.

Conversely, *Helicobacter pylori* ‐positive samples demonstrated lower normalized band areas near 1002, 1154 and 1515 cm^−1^. This combination is compatible with a carotenoid‐associated spectral contribution under visible excitation [38, 39, 40]. The band near 1002 cm^−1^ also overlaps phenylalanine‐related scattering [32]. Lower relative areas do not establish carotenoid depletion or oxidative degradation because normalization, resonance enhancement and mixed molecular contributions affect the measured signal. The 1002 cm^−1^ comparison was the weakest primary result (*q* = 0.0441) and did not reach corrected significance in either site separately. Similarly, the lower normalized area around 2936 cm^−1^ indicates a difference in the C—H stretching envelope, which can contain both lipid and protein contributions [32]. Although *Helicobacter pylori* ‐associated inflammation provides a plausible context for membrane remodeling and metabolic changes [41, 42, 43], this band alone is not specific evidence of lipid loss or peroxidation.

The combination of these relative intensity differences constitutes a candidate spectral pattern of pediatric *Helicobacter pylori* gastritis in this cohort. Statistical support at the patient level strengthens the descriptive comparison, but does not independently validate the biochemical assignments, a disease mechanism or diagnostic performance. Future studies may benefit from integration of Raman spectroscopy with complementary molecular approaches, including proteomics and metabolomics. Proteomic investigations of *Helicobacter pylori* ‐associated gastritis have identified alterations in mitochondrial proteins, oxidative stress pathways, and immune regulatory networks [38, 39, 40, 41, 42, 43, 44, 45, 46, 47]. Combining Raman‐derived molecular fingerprints with multi‐omics approaches data could improve biomarker validation and provide deeper insight into disease mechanisms [32, 40, 41, 42, 43, 44, 45, 46, 47, 48].

From a translational perspective, Raman spectroscopy offers a unique opportunity to characterize these molecular alterations directly in native tissue without staining or extensive sample preparation. Previous adult gastric Raman studies have investigated *H. pylori* infection and intestinal metaplasia [8], providing a rationale for extending spectroscopic investigation to pediatric biopsies. The present study extends these observations to pediatric gastric mucosa and shows that infection‐associated relative spectral differences can be detected in freshly obtained biopsies shortly after collection.

Several limitations should be acknowledged. First, this was a single‐center study conducted in a tertiary pediatric referral setting, which may limit generalizability. Second, the study cohort was relatively small, although the rarity of fresh pediatric gastric tissue suitable for Raman analysis should be considered. Third, only a limited number of measurements could be obtained from each biopsy because of the restricted viability of freshly isolated cells. Additional variability may arise from cellular heterogeneity within gastric mucosa and from residual mucus despite careful sample preparation. Furthermore, two *Helicobacter pylori* ‐positive patients and two *Helicobacter pylori* ‐negative control subjects were receiving proton pump inhibitor therapy at the time of endoscopic examination, reflecting routine clinical practice in children undergoing diagnostic evaluation. Although no obvious spectral clustering according to PPI exposure was observed during exploratory analysis, the sample size was insufficient to evaluate PPI‐associated spectral effects formally.

Despite these limitations, the present study provides the first Raman spectroscopic characterization of pediatric *Helicobacter pylori* gastritis and identifies patient‐level differences in relative Raman intensities associated with infection. These findings support the feasibility of Raman microscopy for investigating gastric mucosal pathology in children and identify candidate molecular signatures that warrant validation in larger independent cohorts.

## 5 Conclusion

Raman microscopy at 532 nm enabled rapid, label‐free molecular profiling of freshly obtained pediatric gastric biopsies and revealed an exploratory spectral pattern associated with *Helicobacter pylori* gastritis. Patient‐level analysis identified differences in the relative areas of all 11 selected Raman bands after correction for multiple testing, with consistent directions of change in the antrum and corpus. Overlapping molecular contributions and normalization‐dependent significance for three bands limit mechanistic interpretation, and diagnostic performance remains to be evaluated. Larger independent cohorts, standardized acquisition and preprocessing, and complementary biochemical measurements are needed to establish the reproducibility and biological meaning of these candidate spectral features.

This study provides a foundation for developing Raman‐based mucosal phenotyping as a potential complement to conventional histopathological assessment of pediatric gastric disease.

## Author Contributions

**Jiří Bufka:** conceptualization, writing – original draft, visualization. **Lenka Vaňková:** conceptualization; writing – original draft, visualization. **Jan Schwarz:** endoscopy, writing review and editing. **Magdalena Daumová:** histology. **Petr Bouř:** writing – original draft; writing – review and editing, supervision. **Josef Sýkora:** writing – original draft, supervision.

## Funding

Charles University, Project No. SVV 260 773, Grant Agency of the Czech Republic (22‐04669S) and the Cooperatio programmes supported this work.

## Ethics Statement

The study was reviewed and approved by the Ethics Committee of the University Hospital Pilsen and Faculty of Medicine in Pilsen, Charles University, on August 31, 2023, with Reference Number 324/23. Informed consent was obtained for the use of the biological materials for research purposes.

## Consent

Informed consent was obtained electronically from all participants included in this study. Participants were provided with detailed information regarding the study's purpose, procedures, potential risks, and confidentiality measures before giving their consent.

## Conflicts of Interest

The authors declare no conflicts of interest.

## Supporting information


**Table S1:** Site‐specific group comparisons. BH correction across all 22 site‐specific tests. Each child contributes one mean per site. Differences concern normalized band areas.
**Table S2:** Group differences in paired site contrasts. Each child contributes antrum minus corpus. Group comparison of this within‐child contrast preserves pairing. BH correction across 11 tests; absence of significance does not demonstrate equivalence.
**Table S3:** Sensitivity to within‐child spectrum weighting. Each child's five or six normalized spectra are averaged directly, instead of assigning equal weights to its two sites. Children remain equally weighted between groups. BH correction across 11 tests.
**Table S4:** Sensitivity to the normalization range. Each spectrum is normalized using the L2 norm of grid points within 600–1800 cm^−1^, then integrated in the original windows and aggregated with equal site weights. BH correction across 11 tests. Scale differs from the primary analysis.

## Data Availability

Results of the supplementary statistical analyses are provided in Supporting Information (Tables S1–S4). The Raman spectral data, patient‐level analytical data, and analysis code supporting the findings of this study are available from the corresponding author upon reasonable request.
