# Supplementary material for: Molecular Profiling of Pediatric Helicobacter pylori Gastritis Using Raman Spectroscopy: Spectral Patterns Corresponding to Local Biochemical Changes
Source: J Biophotonics. 2026 Nov 6;19(11):e70386. doi: 10.1002/jbio.70386 (PMC13743501; doi:10.1002/jbio.70386)
Supplement: Supplementary file 1 [file JBIO-19-e70386-s001.docx]

Supporting Information

Molecular profiling of paediatric Helicobacter pylori gastritis using Raman spectroscopy: spectral patterns corresponding to local biochemical changes

Journal of Biophotonics · Manuscript 4236265

# Patient-level analyses

All analyses use 32 children (12 *H. pylori* positive and 20 controls). The primary estimate averages each child’s antral and corpus values equally. The tables below report the sensitivity analyses defined in Section 2.4.2. Mean differences are infected minus control. CIs are unadjusted 95% Welch confidence intervals. SD denotes standard deviation; BH denotes Benjamini–Hochberg correction.

# Table S1 Site-specific group comparisons

BH correction across all 22 site-specific tests. Each child contributes one mean per site. Differences concern normalized band areas.

| **Site** | **Band (cm⁻¹)** | **Difference (95% CI)** | **p** | **q** |
| --- | --- | --- | --- | --- |
| antrum | 746 | 0.352 (0.171, 0.533) | 0.0009 | 0.0026 |
| antrum | 1002 | -0.102 (-0.225, 0.021) | 0.1013 | 0.1013 |
| antrum | 1126 | 0.422 (0.195, 0.650) | 0.0010 | 0.0026 |
| antrum | 1154 | -0.289 (-0.528, -0.049) | 0.0197 | 0.0241 |
| antrum | 1300 | 0.559 (0.346, 0.772) | 3.28e-05 | 0.0007 |
| antrum | 1337 | 0.601 (0.318, 0.884) | 0.0003 | 0.0022 |
| antrum | 1446 | 0.745 (0.364, 1.126) | 0.0008 | 0.0026 |
| antrum | 1515 | -0.247 (-0.448, -0.045) | 0.0181 | 0.0235 |
| antrum | 1583 | 0.234 (0.088, 0.380) | 0.0028 | 0.0051 |
| antrum | 1657 | 0.411 (0.163, 0.659) | 0.0021 | 0.0047 |
| antrum | 2936 | -1.378 (-1.978, -0.778) | 0.0001 | 0.0012 |
| corpus | 746 | 0.260 (0.126, 0.394) | 0.0007 | 0.0026 |
| corpus | 1002 | -0.147 (-0.298, 0.005) | 0.0573 | 0.0631 |
| corpus | 1126 | 0.273 (0.069, 0.477) | 0.0116 | 0.0159 |
| corpus | 1154 | -0.392 (-0.655, -0.129) | 0.0050 | 0.0078 |
| corpus | 1300 | 0.426 (0.218, 0.635) | 0.0004 | 0.0022 |
| corpus | 1337 | 0.438 (0.203, 0.673) | 0.0010 | 0.0026 |
| corpus | 1446 | 0.529 (0.193, 0.865) | 0.0041 | 0.0069 |
| corpus | 1515 | -0.328 (-0.532, -0.124) | 0.0027 | 0.0051 |
| corpus | 1583 | 0.209 (0.003, 0.415) | 0.0470 | 0.0545 |
| corpus | 1657 | 0.305 (-0.031, 0.641) | 0.0731 | 0.0766 |
| corpus | 2936 | -0.888 (-1.490, -0.285) | 0.0057 | 0.0084 |

# Table S2 Group differences in paired site contrasts

Each child contributes antrum minus corpus. Group comparison of this within-child contrast preserves pairing. BH correction across 11 tests; absence of significance does not demonstrate equivalence.

| **Band (cm⁻¹)** | **Difference (95% CI)** | **p** | **q** |
| --- | --- | --- | --- |
| 746 | 0.092 (-0.085, 0.268) | 0.2818 | 0.3790 |
| 1002 | 0.045 (-0.088, 0.177) | 0.4930 | 0.5423 |
| 1126 | 0.149 (-0.080, 0.378) | 0.1844 | 0.3790 |
| 1154 | 0.103 (-0.100, 0.306) | 0.3088 | 0.3790 |
| 1300 | 0.133 (-0.012, 0.278) | 0.0705 | 0.2303 |
| 1337 | 0.163 (0.007, 0.319) | 0.0414 | 0.2278 |
| 1446 | 0.216 (0.020, 0.411) | 0.0324 | 0.2278 |
| 1515 | 0.081 (-0.080, 0.243) | 0.3101 | 0.3790 |
| 1583 | 0.025 (-0.083, 0.134) | 0.6357 | 0.6357 |
| 1657 | 0.106 (-0.094, 0.307) | 0.2802 | 0.3790 |
| 2936 | -0.491 (-1.053, 0.071) | 0.0837 | 0.2303 |

# Table S3 Sensitivity to within-child spectrum weighting

Each child’s five or six normalized spectra are averaged directly, instead of assigning equal weights to its two sites. Children remain equally weighted between groups. BH correction across 11 tests.

| **Band (cm⁻¹)** | **Difference (95% CI)** | **p** | **q** |
| --- | --- | --- | --- |
| 746 | 0.311 (0.175, 0.447) | 0.0001 | 0.0008 |
| 1002 | -0.134 (-0.259, -0.008) | 0.0375 | 0.0375 |
| 1126 | 0.355 (0.170, 0.539) | 0.0007 | 0.0015 |
| 1154 | -0.354 (-0.590, -0.118) | 0.0046 | 0.0063 |
| 1300 | 0.506 (0.303, 0.708) | 5.30e-05 | 0.0006 |
| 1337 | 0.537 (0.284, 0.789) | 0.0003 | 0.0008 |
| 1446 | 0.652 (0.302, 1.001) | 0.0012 | 0.0022 |
| 1515 | -0.298 (-0.488, -0.107) | 0.0033 | 0.0053 |
| 1583 | 0.217 (0.044, 0.390) | 0.0160 | 0.0196 |
| 1657 | 0.345 (0.062, 0.627) | 0.0188 | 0.0207 |
| 2936 | -1.137 (-1.684, -0.590) | 0.0003 | 0.0008 |

# Table S4 Sensitivity to the normalization range

Each spectrum is normalized using the L2 norm of grid points within 600–1800 cm⁻¹, then integrated in the original windows and aggregated with equal site weights. BH correction across 11 tests. Scale differs from the primary analysis.

| **Band (cm⁻¹)** | **Difference (95% CI)** | **p** | **q** |
| --- | --- | --- | --- |
| 746 | 0.290 (0.065, 0.514) | 0.0140 | 0.0192 |
| 1002 | -0.492 (-0.642, -0.341) | 2.57e-07 | 2.82e-06 |
| 1126 | 0.238 (-0.024, 0.500) | 0.0720 | 0.0880 |
| 1154 | -0.947 (-1.252, -0.642) | 5.73e-07 | 3.15e-06 |
| 1300 | 0.429 (0.254, 0.605) | 3.10e-05 | 8.51e-05 |
| 1337 | 0.463 (0.201, 0.725) | 0.0014 | 0.0025 |
| 1446 | 0.541 (0.201, 0.880) | 0.0034 | 0.0053 |
| 1515 | -0.769 (-1.029, -0.510) | 1.34e-06 | 4.90e-06 |
| 1583 | 0.138 (-0.082, 0.358) | 0.2078 | 0.2286 |
| 1657 | 0.211 (-0.159, 0.581) | 0.2500 | 0.2500 |
| 2936 | -3.741 (-5.873, -1.609) | 0.0012 | 0.0025 |
